# A Novel Forward-Propagation Workflow Assessment Method for Malicious Packet Detection

**DOI:** 10.3390/s22114167

**Published:** 2022-05-30

**Authors:** Nagaiah Mohanan Balamurugan, Raju Kannadasan, Mohammed H. Alsharif, Peerapong Uthansakul

**Affiliations:** 1 Department of Computer Science and Engineering, Sri Venkateswara College of Engineering, Sriperumbudur, Chennai 602117, India; 2 Department of Electrical and Electronics Engineering, Sri Venkateswara College of Engineering, Sriperumbudur, Chennai 602117, India; kannan.3333@yahoo.co.in; 3 Department of Electrical Engineering, College of Electronics and Information Engineering, Sejong University, Seoul 05006, Korea; malsharif@sejong.ac.kr; 4 School of Telecommunication Engineering, Suranaree University of Technology, Nakhon Ratchasima 30000, Thailand

**Keywords:** novel forward propagation, convolutional neural network, k-nearest neighbor, support vector machine, deep learning, machine learning

## Abstract

In recent times, there has been a huge upsurge in malicious attacks despite sophisticated technologies in digital network data transmission. This research proposes an innovative method that utilizes the forward-propagation workflow of the convolutional neural network (CNN) algorithm to detect malicious information effectively. The performance comparison of this approach was accomplished using accuracy, precision, false-positive and false-negative rates with k-nearest neighbor (KNN) and support vector machine (SVM) algorithms. To detect malicious packets in the original dataset, an experiment was carried out using CNN’s forward-propagation workflow method (N = 11) as well as the KNN and the SVM machine learning algorithms with a significant value of 0.005. The accuracy, precision, false-positive and false-negative rates were evaluated to detect malicious packets present in normal data packets. The mean performance measures of the proposed forward-propagation method of the CNN algorithm were evaluated using the Statistical Package for the Social Sciences (SPSS) tool. The results showed that the mean accuracy (98.84%) and mean precision (99.08%) of the proposed forward propagation of the CNN algorithm appeared to be higher than the mean accuracy (95.55%) and mean precision (95.97%) of the KNN algorithm, as well as the mean accuracy (94.43%) and mean precision (94.58%) of the SVM algorithm. Moreover, the false-positive rate (1.93%) and false-negative rate (3.49%) of the proposed method appeared to be significantly higher than the KNN algorithm’s false-positive (4.04%) and false-negative (6.24%) as well as the SVM algorithm’s false-positive (5.03%) and false-negative rate (7.21%). Hence, it can be concluded that the forward-propagation method of the CNN algorithm is better than the KNN and SVM algorithms at detecting malicious information.

## 1. Introduction

In today’s world, fully submerged in the digital era, much advancement has been made in technologies related to the network transmission of data, including video, audio, and text. Networks have been enhanced as per the sophistication of users and their needs. These networks can be classified as cloud computing, fog computing, grid computing, wireless sensor networks (WSN), Internet of Things (IoT), etc. Data can be transmitted over these networks either secretly or openly based on the size of the data and the types of applications in use. Today’s sophisticated digital world has necessitated the introduction of newer technologies and user-friendly devices, which in turn have enormously impacted the areas of research and business. At the same time, there has been a surge in the number of threats injected as attack patterns into malicious packets to gather valuable and secret data over the internet [1]. To counter this, authenticated transmissions have been introduced using various cryptographic algorithms and authentication mechanisms to detect or offset malicious packet patterns. In recent times, deep-learning algorithms have played an inevitable role in the smart detection and identification of malicious packets in network transmissions. One of the key works related to this is the behavior-based anomaly detection in traffic patterns, which was proposed by Karasek et al. [2] and Thamilarasu et al. [3]. The authors proposed a model using a deep-learning algorithm to detect malicious behaviors by checking users’ time-series data from network packets. The trained model defined all aspects of network behavior. The model’s accuracy was compared by testing the first five end-to-end conversations and was reported to have achieved 95% accuracy and a 93% recall rate. Datasets were used from live network data [4,5]. False positives were tested and evaluated as 1.1%, with the execution time being 85.20 milliseconds for each conversation. In addition, the identification and tracking of malicious nodes in the network were addressed. This review studied various approaches comprising deep-learning algorithms to identify malicious packets and malicious nodes.

The identification of malicious packets is crucial for safeguarding sensitive data communications in the modern digital age and for creating a user-friendly and authenticated environment. This dangerous trend of malicious packet transmission over networks has only been increasing with time, and an effective counter-application to be adopted across all network traffic is the need of the hour. Since traffic patterns could be irrevocably compromised in networks with the injection of such malicious information, many malicious packet identification techniques have been proposed as counteractive measures [6,7,8]. Shinan [9] proposed a botnet detection procedure with the help of software-defined networking (SDN), supervised learning algorithms and a flow-based detection technique. Very timely and accurate botnet detection has been achieved using machine learning algorithms. This technique has interfered with botnet traffic by effectively evaluating network traffic and detecting malicious information. The advantage of this technique is the number of packets per flow and the limited time periods for which traffic flow has to be monitored in order to achieve accurate detection.

There is a gap concerning the existing CNN, KNN, and SVM algorithms. Obtaining relevant performance measures has been a challenge, and there is a need for higher accuracy and precision as well as reduced false-positive and false-negative rates in detecting malicious packets in standard packets. Furthermore, the need for adding more values to the dataset and training the dataset for more accurate predictions cannot be emphasized enough.

The following sections are outlined in the research: Section 2 discusses related works utilizing CNN, KNN and SVM algorithms and other machine-learning and deep-learning algorithms. Section 3 describes the novelty of the proposed work and its working mechanism and performance parameters and related terminologies. The result of the research is explained in Section 4. Section 5 discusses the novelty of the research along with its limitations. Finally, Section 6 describes the conclusion and possible extensions in the future.

## 2. Related Work

Some authors have proposed anomaly and network intrusion detection mechanisms using machine-learning algorithms, discussed by Gilmore et al. [10]. This research intends to predict malicious information present in a dataset of network packets with better accuracy, precision, false-positive and false-negative rates using CNN, KNN and SVM algorithms. Table 1 lists the methods and approaches of malicious packet identification that have been used in various works.

Several works have been reported by the authors related to malicious packet detection. Thamilarasu et al. [3] proposed a technique for detecting intrusion within an internet environment using DL algorithms. In this work, an intelligent intrusion detection system was adapted to an IoT environment. This system detected malicious traffic in an IoT network using deep-learning algorithms. This mechanism was also evaluated for interoperability within various communication networks and providing security as a service (SaaS) to the networks. A real network trace was carried out to evaluate the proposed framework’s effectiveness and trace its stability. It was found to be perfect for real-world intrusion detection in actual network data transmissions, but a drawback is that this solution is only concerned with security. In this context, some of the prevalent applications for malicious packet detections are discussed as follows. Ren-Hung Hwang et al. [11] proposed a solution based on packet-level-based malicious traffic classification using a deep-learning approach called a short-term long memory (LSTM). In this work, a solution for reducing flow-based data processing was evaluated, which enables hackers to exploit the delay time in the network traffic. This work was duly enhanced to reduce the data-processing flow and detect real-time malicious traffic. Deep learning was used to train and test the malicious packets. A DL-based LSTM model with word embedding as a mechanism was devised. The word-embedding mechanism was extracted from the packets’ header fields and checked for normal or malicious traffic. The packet datasets were collected from the IoT of Mirai Botnet, USTC-TFC2016, and ISCX2012 of Robert Gordon University [11]. As a result of this work, IDSs were found to work without delay in identifying malicious packets. 

Cheng [1] proposed a deep-packet inspection method using CNN. This work was used to identify the malware by penetrating network traffic. The CNN and character embedding were used in the mechanism, and payloads of packets from the trained dataset were analyzed. The total malware datasets used were 127, and the sample of payload traffic was around 16 GB. The proposed model acted as the NIDS and was compared with the Snort IDS and was found to achieve 15% more in the F1 score (F1 score is defined as the harmonic mean of precision and recall). The D1 is actually model accuracy measured in the dataset. This work only used 127 malware datasets. Yet another work for penetrating malicious packets in vehicle ad-hoc networks (VAN) was proposed by Kang et al. [12] using deep neural networks (DNN) and coined the intrusion detection system (IDS). The probability of discriminating information from VAN packets was checked to determine whether they were normal packets or attack packets. A deep belief network (DBN) was coined using the DNN unsupervised algorithm, and it was pre-trained for network packet parameters. The DBN was found to achieve improved accuracy in real-time traffic. Based on all of the research that has been carried out on the use of malicious packet identification algorithms, deep-learning algorithms such as CNN [1] and DNN [12] have been found to be the best. However, this work can only solve packets of VAN. In another instance, signature-based malware packet detection was carried out by Ahmed et al. [13]. This research claimed that there should be mechanisms to identify variants of the malware packets in the network. The malware packets are usually introduced in traffic like YouTube, bloggers, and online shopping sites. This work identified the malware packets in two steps. The first step checked whether the packet was a multimedia type. The second step classified the packets as text/executable formats. The parameters evaluated were false-negative (4.69%) and false-positive rates (2.53%). Finally, a simple statistical analysis was performed on the evaluation parameters. This work was only concerned with multimedia datasets.

Concerning network malicious packet checking, several mechanisms have been proposed. One such mechanism dealt with network-based anomaly detection [6]. It used deep auto-encoders on the network traffic to check for malicious packets. The IoT traffic was used as a dataset. Bashlite botnets were used to infect the IoT device with malicious packets. Performance was measured with respect to the diversity of the data exchange. It was time-consuming work. In another instance, a CNN-based mechanism [14] was introduced to classify malware traffic from normal traffic. It was the only learning approach used for malware, and the dataset used consisted of traffic images. This mechanism was found to be lacking in the detection of unknown attacks and also took more detection time to classify malicious attacks. A recurrent neural network (RNN) model [7,8] was similarly proposed to train raw packets and extract micro-flow features from raw packets for small sets of packets. This work solely concentrated on accuracy, but the time taken to trace the packets was high. Yet another study was carried out to detect malware packets with regard to accuracy and false positives. Two algorithms were used to detect the network traffic’s executable files (malware). The dataset used to penetrate the packet was VXHeaven [15]. All of the work related to malicious packet and malicious traffic identification methods and their application is listed in Table 2. Most of the work related to malicious packet identification was coined and discussed using machine-learning algorithms like KNN and SVM [16]. 

Other work related to malicious malware detection was carried out using the CNN algorithm [17] by identifying selected packet fields and port numbers. This work aimed to segregate unpredictable port numbers and protocols, addressing as many as 35 features of the packet. The result was also compared with SVM and random forest (RF) algorithms. The limitation of this work was that it only used 31 features of the packet, even while using the CNN algorithm to predict more accurately. The parameters used to compare the different algorithms were accuracy, precision, and recall.

De Lucia et al. [18] proposed work related to malicious information identification. Here, an approach was coined to identify the encrypted malicious traffic from the network-transmitted data packets. Since machine learning was essential in detecting malicious traffic from real-time networks, this work used SVM and CNN algorithms. The reason for introducing these algorithms was that usually, malicious users use transport layer security (TLS) to hide the malicious information [19,20]. The experiment was conducted to check performance related to detecting malicious information. The false-positive rate was used as a parameter to check the performance of the machine-learning algorithms. The limitation of this work was that the encrypted malicious packet detection took more time than normal malicious packet detection mechanisms. 

Marín et al. [19] proposed a mechanism for malware traffic detection and classification using deep learning and block chain security for mutual authentication. A deep model was framed from deep learning on specific raw traffic feature representation problems [20]. This feature representation included low level and packet level. This deep model detected the statistics of malicious traffic without including handcrafted features. The experiment detected malicious traffic with better accuracy than normal malware traffic detection. The limitation of this work is that the proposed model could take more time to predict malicious patterns. Malware traffic detection was also discussed for machine learning and the challenge to detect malware by Ronen et al. [21], Shone et al. [22], Sharafaldin et al. [23] and Kim et al. [24].

Research into web anomaly detection using the SVM algorithm was also discussed by Erfani et al. [25]. The limitation of this work was a high false-positive rate. Another study related to attack fingerprinting using a naïve Bayes (NB) algorithm was discussed by Herrmann et al. (2009) [26] for privacy enhancement, but the accuracy of this work was found to be considerably less. Reddy et al. [27] similarly proposed work related to attack detection and mitigation and framed an SaaS-based model. This work was compared with the SVM and NB algorithms and was found to achieve only limited accuracy. Further work that addressed encrypted malicious detection was discussed by Lotfollahi et al. [28]. Riyaz et al. [4] proposed a deep-learning solution for effectively detecting intrusion information using the CNN algorithm. This approach was used to detect intrusion in communicated data over WSN. The classification and features were extracted from the CNN algorithm’s communicated data and achieved 98.88% accuracy. The work was cross-validated with tenfold cross-validations to check performance. In another example, work that detected Botnet attacks with a machine learning algorithm was coined by Soe et al. [20]. This work overcame the rule-based detection of attacks in an IoT environment. A machine-learning-based sequential architecture was proposed here. This sequential architecture was framed using three machine-learning algorithms: artificial neural network (ANN), NB, and decision tree algorithms. An experiment was conducted to detect Botnets. In this work, the ANN proposed max–min normalization in order to detect Botnets, and it is represented as Equation (1), which can be used for preprocessing in the ANN. The eight sub-engines utilized were ack, junk, scan, udp, tcp, uplain and combo.

(1) $$a=\frac{\left(b\right)}{\left(b\right)-min\left(b\right)}\;$$

where “*a*” is a normalization factor, and “*b*” is the input to the normalization function. The activation function used in this ANN algorithm is represented as Equation (2).

(2) f(x)=(0,x)={x,ifx≥0∨0,ifx<0}

While many studies have addressed the detection of malicious packets in network transmission, there are still challenges in this area regarding speed, accuracy and precision, which pose a significant threat to cyber society, especially in a world inundated with increased network traffic. Therefore, it is essential to overcome this threat by introducing more relevant and accurate mechanisms for handling malicious packet identification. This work has sought to make a research contribution in lieu of a proposed CNN-based forward-propagation workflow method to analyze and detect malicious packets more accurately and deeply. Experiments have been conducted using a Python deep learning package-based algorithm and a malicious packet dataset. Subsequently, the results have been compared with the KNN and SVM algorithms to measure the accuracy.

## 3. Proposed Methods

This research was carried out in the Department of Deep Learning, Saveetha School of Engineering, SIMATS, Chennai. The number of groups used was three: one was for the forward-propagation workflow of the CNN algorithm, and the other two were for the KNN and SVM algorithms. The transmitted packets used were referred from the dataset [22], and USTC-TFC2016 and ISCX2012 of Robert Gordon University [11] with 141,000 records and 61 fields, also called features of the packets. In total, 80% of the records were utilized for training and 20% for testing. For each algorithm, 11 sample iterations were performed, the G power was calculated as the actual power of 80% and the alpha value was set at 0.005. All three algorithms were used and analyzed for malicious information from the dataset. In the experiment conducted, from the total number of dataset records, the forward-propagation workflow of the CNN algorithm found a total of 97,453 malicious packets, while the rest of the count comprised genuine packets. 

**Figure 1:**
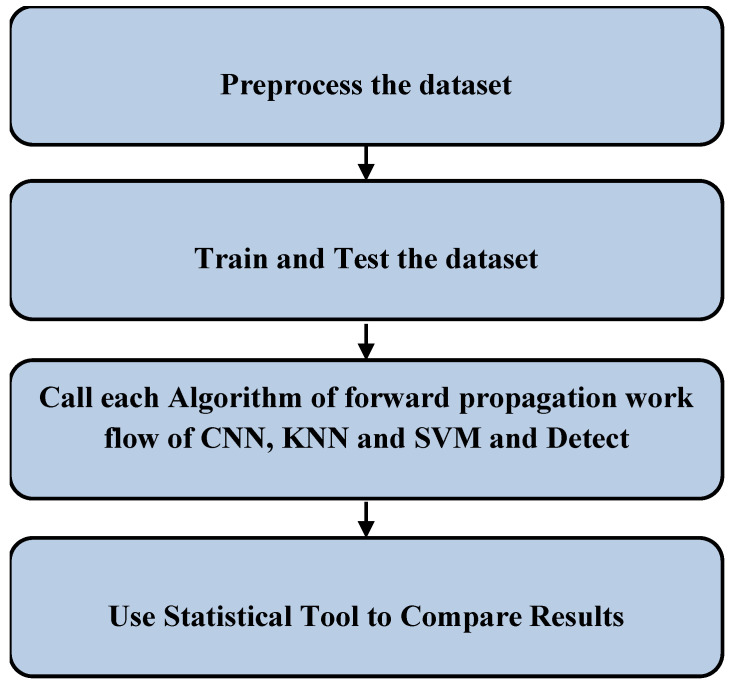
Malicious information detection steps.

Figure 1 shows the mechanism that was carried out to trace the malicious information from packets based on the dataset. Initially, 61 fields of records were preprocessed. Later, trained and testing datasets were assigned. The algorithms were used on the training and testing datasets for detecting malicious information, and the performance measures were noted. Using the SPSS statistical tool, the mean accuracy, mean precision, mean false positive and mean false negative were analyzed concerning the forward-propagation workflows of the CNN, KNN and SVM algorithms.

### 3.1. Accuracy and Precision for Malicious Packets

**Figure 2:**
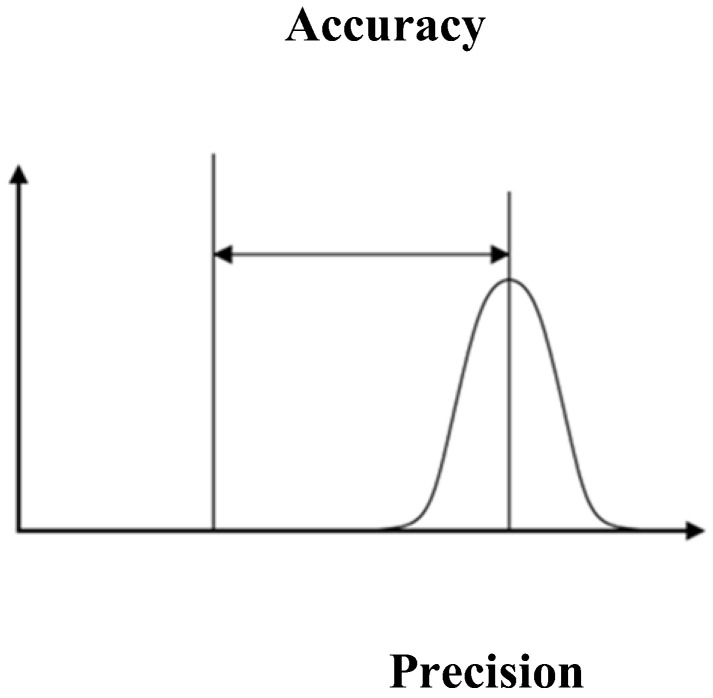
Illustration of Accuracy and Precision Parameters.

The accuracy of identifying malicious packets rests on the closeness of the specific value, while the precision is the measurement of the proximity of the packets to each other while checking for malicious packets. Figure 2 illustrates the accuracy and precision parameters. Equation (3) represents the accuracy of malicious information identification.

(3) $$Accuracy=\frac{TP+TN}{TP+TN+FP+FN}$$

where *TP* is true positive, *TN* is true negative, *FP* is false positive and *FN* is false negative. Equation (4) is a formula that is used to measure the precision of malicious information.

(4) $$Precision=\frac{TP}{TP+FP}$$

### 3.2. False Positive and False Negative for Malicious Packets

Table 3 illustrates the false-positive and false-negative information. False positives are those in which the packet was not malicious, but the detection technique identified it as malicious information. When the packets are traced for malicious content, if the packet is malicious and the identification of the packet finds it malicious, then it is a true positive. On the other hand, true negative is the one in which the packet actually did not contain malicious information, and the detection also identified it as not malicious. Finally, a false negative is nothing but the detection technique judging that no malicious events were found while checking existing malicious packets. 

Equation (5) was used to measure the false-positive rate (*FPR*), and Equation (6) was used to measure the false-negative rate (*FNR*) for malicious information detection from the packet dataset.

(5) $$FPR=\frac{FP}{FP+TN}$$

(6) $$FNR=\frac{FN}{FN+TP}$$

### 3.3. Working Mechanism of CNN

**Identification by CNN (Sample Preparation Group 1):** In this model algorithm, CNN classification was divided into three steps [4,29,30]. 

**Training of dataset records:** This step aimed to train the 62 dataset fields to adopt the trained model. In order to evaluate the process, it was analyzed for accuracy, precision, false-positive and false-negative parameters. In order to enable the proper identification of malicious information from the packet dataset, a number of records from the dataset had to be tested with the initial layers, while the remaining layers were released.

**Model Optimization:** To strengthen the model’s performance metrics values, additional settings of the augmentation process were carried out. The last two layers were trained at the set evasion training rate. 

**Identification:** Finally, the interpretation process was set based on the training and test datasets. Additionally, the identification function was created in order to identify and achieve accurate, precise and reduced false rates for positive and negative. 

**Figure 3:**
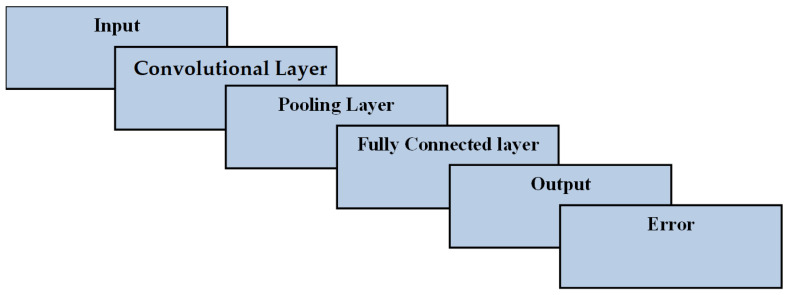
The CNN algorithm’s layered workflow for input–output.

In the CNN algorithm [31,32,33], three layers were used: the convolutional layer, the pooling layer and the fully connected layer. The convolutional layer was used to extract features from the dataset packet records as inputs and as the filtering process for producing the outputs. The pooling layer was a pooling operation to reduce the dimensionality of the feature extraction. The fully connected layer was a connected layer and a multi-layer perceptron along with activation functions at the output layer. Figure 3 illustrates the workflow of the CNN layers along with input, output and error. Usually, CNN works well for forward and backward propagation. The CNN algorithm uses forward propagation to produce outputs from the inputs, after processing and crossing all of the layers. Sometimes, the output does not meet the threshold and makes an error. In that case, it uses backward propagation to train the input layer, including the hidden layers, in order to achieve proper outputs the next time. 

In general, CNN uses the following output expression at the output end and is represented as Equation (7):

(7) d=e*f()

where “*d*” is the output expression, “*e*” is the input to the CNN algorithm for malicious information identification and f() is a function for extraction filters. The confusion matrix of the fully connected layer enables the feature extraction of linear transformation. The following Equation (8) represents the feature extraction of linear transformation for malicious information identification with 61 features of records of packet information.

(8) $$d=h^{t}\cdot i+j$$

where *g* is the feature extraction of linear transformation for malicious information identification, *h* is the weight of input, *i* is the input, *j* is the bias threshold and *t* is the transformation. The workflow of forward propagation is laid out in the following section. 

### 3.4. Forward-Propagation Work Flow

**Figure 4:**
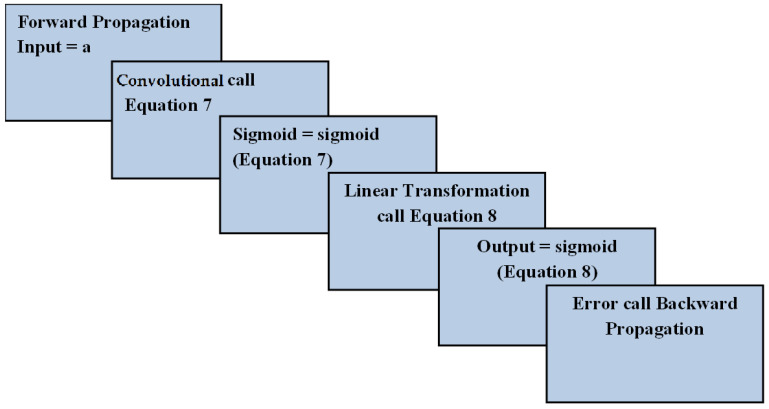
Workflow of forward propagation.

The CNN forward propagation was assigned an input, say “a.” The convolution step called for Equation (7) to extract malicious information. Next, the sigmoid threshold function called for Equation (7). The linear transformation of malicious information was called once the threshold function was computed. The sigmoid threshold function was again called to feed the linear transformation as the input for Equation (8). Finally, the output was predicted based on the second iterated threshold sigmoid function. In case of an error, the backward propagation was called. The workflow of the forward propagation is depicted in Figure 4, which illustrates its novelty in detecting malicious information.

### 3.5. Identification Mechanism of the KNN Algorithm for Malicious Information

In the method called KNN_classifier, the input was set as input_data, the run for the feature was set, and the labels of records, n_neighbor, were set to 1. The n_neighbor was run in a loop up to the range. The weight was calculated based on distance and uniform. The metrics for Manhattan and Euclidean distance were calculated. Then, the loop was made to run from metric till metrics with inner for loop weight in weights. The classifier was calculated with the Kneighbor_classifier method with the parameters as n_neighbor and metric and weight from the loop variable. The loop was then called for run_for_features. From this loop, the k features were logged. The best_features _data was calculated by keeping selectKbest method as the input_data and the data labels as parameters. The X_training, X_test and Y_Test were calculated from the train_test_split method with parameters such as labels, test_size and best_features_data. Finally, log.info() method with parameter weight and metric as a parameter was calculated, followed by the classifier_fit() method with X_train and Y_train parameters. In this manner, the score was calculated with the classifier_score() method with the test parameters for X and Y. 

The next step of the KNN algorithm measured the confusion matrix using the classifier_predict() methods with X_test and was stored in the variable result followed by conf_matrix() method, along with Y_test and result parameters. The accuracy score was called, and precision was calculated with the conf_matrix current value with the neighboring value. The recall value was also calculated with the conf_matrix method. Finally, the false-positive and false-negative parameters were also calculated using a confusion matrix. Subsequently, performance parameters like accuracy, precision, false positive and false negative were called for 11 iterations using Equations (3)–(6). Based on the KNN algorithm, graphs were plotted with the SPSS statistical analysis tool for all four performance-measuring parameters. The comparison of the performance parameters was carried out with the forward-propagation workflow of the CNN algorithm for the said performance parameters. 

### 3.6. Malicious Identification by SVM Algorithm

Once the training and testing were initialized, the SVM algorithm, SVC classifier list and SVC feature list were assigned along with the tree selection list and the feature called. The data were initialized with dependent variables and assigned with the stack method to obtain the confusion matrix. Then, the average matrix was calculated with the average matrix and dependent variable. The heatmap method was called and represented the confusion matrix for malicious and normal packets, and finally, the confusion matrix was plotted with the cmap parameter. If a matrix got the linearSVC, then the matrix was appended with the SVC list. In the case of tree selection, the tree selection list was appended; otherwise, it was appended to the non-feature list. Next, this procedure called the shown graph for the SVC with parameter classifier and features. After that, the train decision tree method was called along with the X and Y axis, and the test size was set between 0 and 1. The transform method was called to decide about the packet identification. This method checked the keywords to determine whether they were malicious or not. It dropped genuine keywords if necessary. Finally, it showed the average accuracy and training time. After this scheduled process, the confusion matrix was drawn with accuracy. The same procedure was then followed for precision, false positive and false negative by calling Equations (3) to (6).

### 3.7. Testing Procedure for the Proposed Work and Analysis

The train and test percentage of the data were calculated from the dataset. The accuracy, precision, false positive and false negative were represented as the lost modes that should be found. The general architecture of the CNN was depicted with the connected nodes and the arrows; each arrow connected nodes from input to output with its weight and kept it at the output node. The test was trained with 5, 10 and 25 epochs. During the initial state, the knowledge gain for the forward-propagation workflow of the CNN was set as 0.01. The dataset with all records was checked using the forward-propagation workflow of CNN, KNN and SVM for measuring the accuracy, precision, false positive and false negative. The analysis tool SPSS was used to compare all of the algorithm performance measures. Three independent variables were taken: variable 1 was set as the forward-propagation workflow of CNN, variable 2 was set as KNN, and variable 3 was set as SVM. Four dependent variables were taken and these variables were considered in the analysis procedure. Analysis was carried out for the accuracy, precision, false positive and false negative, and all of the measures were plotted as bar graphs with mean, standard deviation and error rates. The descriptive statistics were applied using the SPSS statistics tool. 

## 4. Experiment Results 

### 4.1. Performance Comparison of CNN and KNN Algorithms

The malicious information identification experiment was carried out using the forward-propagation workflows of the CNN, KNN and SVM algorithms on the network packet dataset. This was followed by the statistical analysis performed to find the mean accuracy, mean standard deviation (SD) and the mean error. The graphs were plotted for the forward-propagation workflows of CNN, KNN and SVM for mean accuracy, mean precision, mean false positive and mean false negative. The following tables and figures explain the inference of the experiment in detail. The graphs were plotted to compare the number of iterations for the forward-propagation workflows of the CNN and KKN, initially with four performance measuring parameters. The parameters were accuracy, precision, false positive and false negative. Table 4 lists the performance metric values of the 11 iterations for the forward-propagation workflows of both the CNN and KNN algorithms. 

Table 5 illustrates the group statistics for the performance measures. Eleven iterations were carried out for the forward-propagation workflow of the CNN and KNN algorithms. The inference from Table 1 is that the mean accuracy was 98.85% and 95.55% for the forward-propagation workflow of the CNN and KNN algorithms, respectively. The mean precision was 99.088% and 95.97%, respectively, for the forward-propagation workflow of the CNN and KNN algorithms. It was usually observed that the identification was improved with increased precision. The third parameter was false positive; here, 1.93 and 4.04 were the metrics for the forward-propagation workflow of the CNN and KNN algorithms, respectively. Finally, the false-negative mean values were 3.49 and 6.24, respectively, for the forward-propagation workflow of the CNN and KNN algorithms. 

Table 6 lists Levene’s test for equality of variances and t-test for equality of means for performance-measuring parameters. For 11 epochs, the significant values were produced with the SPSS tool’s statistical analysis for the performance comparison of the forward-propagation workflows of the CNN and KNN algorithms. The accuracy compared to the significant value produced was 0.047. The precision compared to the significant value was 0.138. The false positive compared to the significant value was 1.201. The false negative compared to the significant value was 2.175. The inferences from the statistics are as follows. The significant values of the forward-propagation workflow of the CNN accuracy and precision were better than the KNN accuracy and precision. However, the forward-propagation workflow of the CNN false positive and false negative appeared to be significantly better than the KNN false positive and false negative. 

**Figure 5:**
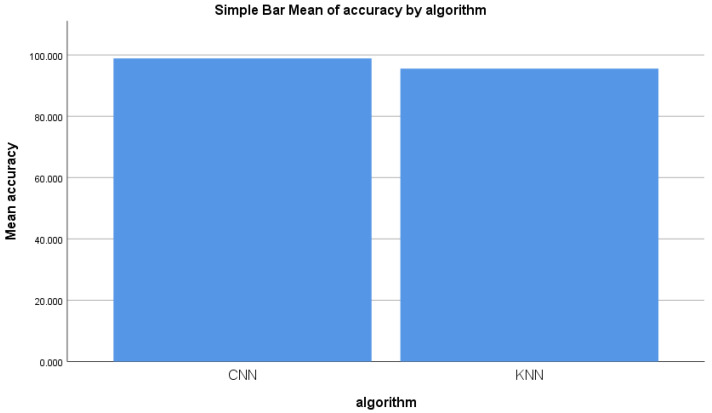
Accuracy mean for CNN and KNN performance measure.

From the experiment conducted for identifying malicious information, the mean accuracy was calculated statistically. The mean accuracy of the forward-propagation workflow of the CNN algorithm was determined to be 98.848% after 11 epochs, with a maximum accuracy of 99.984% and a minimum accuracy of 97.644%. The KNN algorithm’s mean accuracy was statistically determined to be 95.551%, with a maximum accuracy of 96.866% and a minimum accuracy of 94.68%. Figure 5 illustrates the mean accuracy of both algorithms. The accuracy of the performance of the forward-propagation workflow of CNN as a means of identifying malicious information was found to be more than that of the KNN. Figure 5 illustrates the mean accuracy comparison of both the CNN and KNN algorithms. 

**Figure 6:**
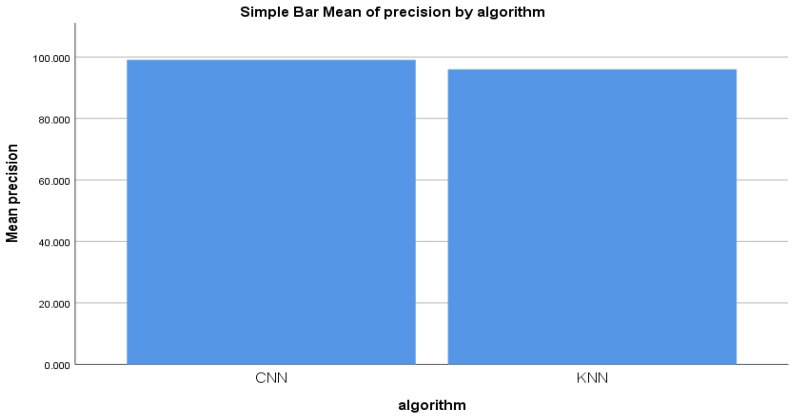
Precision mean for the CNN and KNN algorithms’ performance measure.

From the comparison experiment of the forward-propagation workflow of the CNN and KNN algorithms, the mean precision was also compared. The figure illustrates the comparison of mean precision. A statistical difference was observed between the comparison of the mean accuracy of the forward-propagation workflow of the CNN and KNN algorithms. Figure 6 illustrates the graphical difference between the CNN and KNN algorithms.

**Figure 7:**
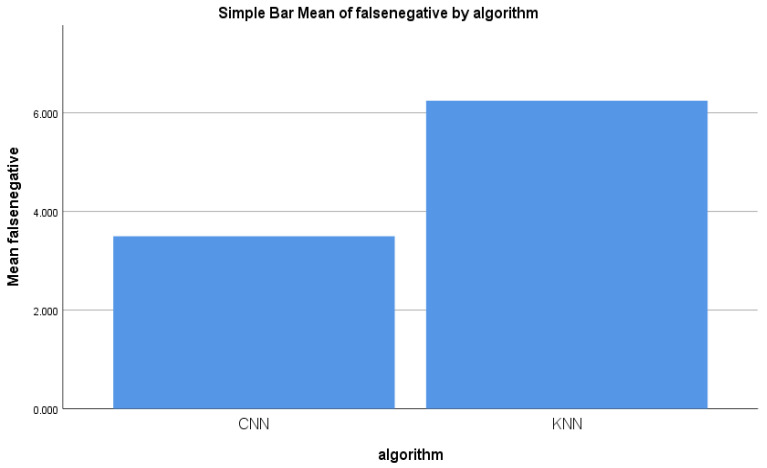
False negative mean for the CNN and KNN algorithms’ performance measure.

Similarly, the other two performance parameters that checked for malicious information records from the dataset were false positive and false negative. Figure 7 illustrates the comparison of the mean false negative. A considerable difference was observed between the false negative of the forward-propagation workflow of the CNN algorithm and the false negative of the KNN algorithm. Figure 7 shows the difference in the mean false negative between the two algorithms. In general, a smaller % of false negative indicated better accuracy.

**Figure 8:**
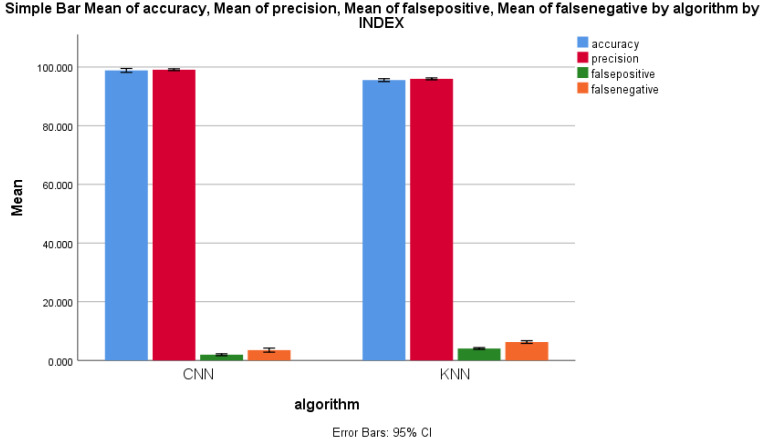
Mean accuracy, mean precision, mean false positive, and mean false negative of CNN and KNN algorithms’ performance measure with ±1 standard deviation.

The performance parameters of mean accuracy, mean precision, mean false positive and mean false negative were measured, and the same were plotted on a graph in order to compare the error rates of the forward-propagation workflows of the CNN and the KNN algorithms. Figure 8 shows the performance measure comparison of both algorithms. It clearly shows a considerable difference between all of the performance measures of both forward-propagation workflows of CNN and KNN algorithms. For 11 epochs, the performance measures for the CNN algorithm were always higher than those of the KNN algorithm, with an increase in the sample size. The standard deviation of the forward-propagation workflow of the CNN algorithm was slightly higher than that of the KNN algorithm for all performance measures for malicious packet record identification, which is illustrated in Figure 8. The X axis represents the CNN vs. KNN algorithms. The Y axis represents the mean accuracy comparison of the forward-propagation workflows of the CNN and KNN algorithms with ±1 standard deviation.

**Figure 9:**
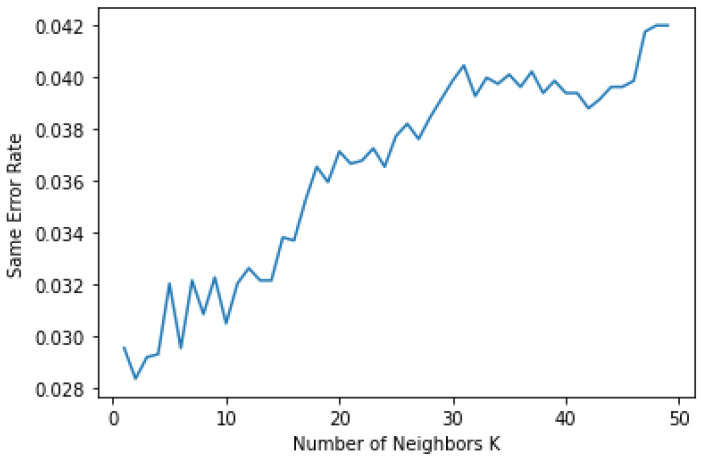
Linear growth of same error rate in KNN algorithm.

Figure 9 illustrates the same error rate of the KNN algorithm for the number of epochs. It can be observed that if the sample and epoch size increased, then an identical error also increased in linear order. Here, the X axis represents the number of epochs of the KNN algorithm, and the Y axis represents the error rate with *p* < 0.05.

### 4.2. Performance Comparison of CNN and SVM Algorithms

The experiment was conducted to measure the performance of the forward-propagation workflow of the CNN and SVM algorithms for malicious information identification from the packet dataset. Table 7 shows the performance measures of the forward-propagation workflow of the CNN and SVM algorithms, namely, accuracy, precision, false positive and false negative, for 11 epochs. The accuracy of the CNN algorithm was observed to be 99.894% and 97.644%, respectively, as the maximum and the minimum. For the SVM algorithm, the maximum accuracy attained was 95.747, and the minimum was 93.561%. There were considerable deviations observed in the accuracy, precision, false positive and false negative concerning both the CNN and SVM algorithms.

Table 8 lists the mean accuracy, mean precision, mean false positive and mean false negative of the 11 epochs of the forward-propagation workflow of the CNN and SVM algorithms for detecting malicious information. The mean accuracy of the CNN algorithm was found to be 98.84%, whereas, in the case of the SVM algorithm, it was found to be 94.43%. The mean precision of the CNN algorithm was found to be 99.09, whereas, in the case of the SVM algorithm, it was found to be 94.59%. The inference is that there was a considerable significant difference between the algorithms for accuracy, precision, false positive and false negative. 

Table 9 shows the comparison of the parameters of performance measure from the independent samples test concerning the forward-propagation workflows of the CNN and SVM algorithms. This table was constructed to check the differences between the two algorithms employing Levene’s test for equality of variances and the t-test for equality of means. From this table, it can be inferred that there was considerable significant variation for accuracy, false positive and false negative for the CNN and SVM algorithms, but there was a slightly significant difference between CNN and SVM precision. There was also ±1 standard deviation concerning the CNN and SVM algorithm performance measures. 

**Figure 10:**
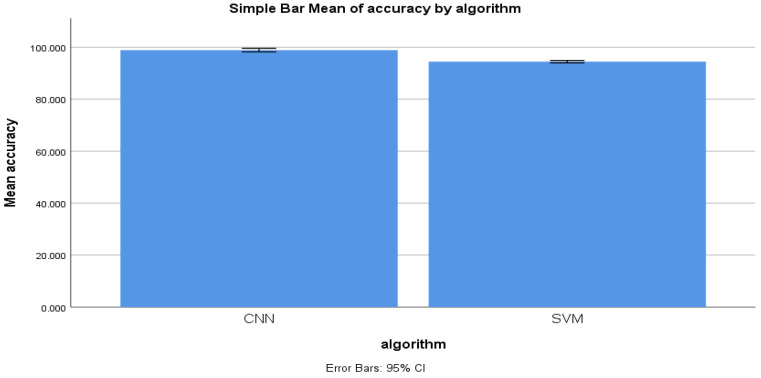
Simple mean accuracy for CNN and SVM algorithms.

Figure 10 shows the mean accuracy of the CNN and KNN algorithms with error rates of 11 epochs. There is a significant difference between the mean accuracy of both algorithms. 

**Figure 11:**
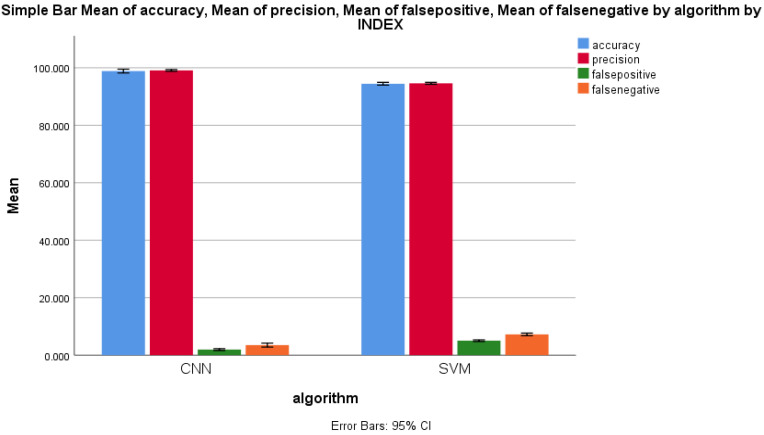
Mean accuracy, mean precision, mean false positive, and mean false negative of the CNN and KNN algorithms’ performance measure with ±1 standard deviation.

Finally, the performance measures of all four parameters were compared with the standard error rate for the forward-propagation workflows of both the CNN and SVM algorithms. Figure 11 illustrates the difference between these two algorithms. It is clear that the F value, mean difference, standard error difference and the 95% confidence interval of difference were also considerably dissimilar between the CNN and SVM algorithms. Figure 11 illustrates the performance comparison of both algorithms with respect to the mean SD and mean standard error. Accuracy is not directly related with either false positive or false negative, but with respect to TPR and FPR, there is a relationship. Likewise, there is an indirect relationship between the false positive and false negative.

## 5. Discussions

A statistical test was conducted as per reference [34]. The performance parameters’ experimental values were measured and noted for different iterations. It was observed in this research that the accuracy, precision, false positive and false negative with respect to malicious packet detection were better when using the forward-propagation workflow of the CNN algorithm rather than that of the KNN and SVM algorithms. The forward-propagation workflows of the CNN, KNN and SVM algorithms were made to undergo iterations with a sample size of 650. Eleven epochs were iterated for detecting the accuracy, precision, false-positive and false-negative percentages concerning malicious packet detection. The dataset in question contained 141,000 records and 61 fields, with 80% of them trained and 20% tested for experiments conducted in order to compare the performance measures of the CNN, KNN and SVM algorithms. It was observed from the internal computations that the forward-propagation workflow of CNN consumed less storage than that of the KNN and SVM algorithms. The accuracy rates for malicious content detection were 98.84855%, 95.55198% and 94.43319%, respectively, for the CNN, KNN and SVM algorithms, and as a result, it was concluded that the mean accuracy for the CNN algorithm was better than that of the KNN and SVM algorithms. 

The malicious content detection precision rates were observed to be 99.08827%, 95.97619% and 94.58727%, respectively, concerning the forward-propagation workflows of the CNN, KNN and SVM algorithms, and as a result, it was concluded that the mean precision for the CNN algorithm was significantly better than that of the KNN and SVM algorithms. Likewise, the false positive and false negatives were also found to be considerably better for the CNN than the KNN and SVM algorithms.

The confusion matrix was plotted in order to undertake investigations with respect to the detection rate. Graphs plotted for 11 epochs helped observe that the mean accuracy and the mean precision were in linear order. The standard mean for the algorithms was calculated from 11 iterations, and subsequently, the standard deviation and mean standard error for all three algorithms were tabulated. It was concluded that with a significant difference of *p* < 0.05, the algorithm produced an effective output. 

While comparing the accuracy, precision, false positive and false negative of this work with respect to existing studies [1,2,9,35], it was observed that there was a considerable improvement in the performance of all parameters. With an increase in the number of samples of work from those undertaken by [6,7], linear growth in accuracy was observed in the CNN algorithm. In some other algorithms, for example, in the recurrent neural network (RNN) [8,35], the accuracy was similarly improved at a linear rate. In subsequent works, live network traffic analysis can be conducted with specifically prepared datasets, as discussed in network anomaly detection works [36]. The applications of malicious packet detection are secure authentication and communication [37,38], reliable blockchain-based applications with signature authentication for health care IoT [39,40] and blockchain-based secure cloud storage [41]. 

Many factors affect malicious packet identification, including the header fields of the packets and the dataset size. There is a requirement for a higher confusion matrix transformation to be worked out. The limitation of the forward-propagation workflow of CNN was the slightly higher time complexity compared to SVM. A persistent challenge is that an increase in the number of fields in the dataset record leads to a corresponding increase in the rate of time consumption [26,27]. If the numbers of iterations are increasing, performance parameters’ values are increasing accordingly. Obviously, SVM and KNN have been used for a decade, but many DL-based and unsupervised learning algorithms work efficiently and give better results.

## 6. Conclusions and Future Work

The intent of the experiment was to implement the forward-propagation workflow of the CNN, KNN and SVM algorithms to detect malicious packets from dataset packets and to compare the performance of the algorithms concerning accuracy, precision and false-positive and false-negative rates. The CNN algorithm’s mean accuracy (98.84855%) and mean precision (99.08827%) were found to be higher than the KNN algorithm’s mean accuracy (95.55198%) and mean precision (95.97619%), as well as the mean accuracy (94.43319%) and mean precision (94.58727%) of the SVM algorithm. The false-positive (1.93287%) and false-negative (3.49736%) values in the CNN algorithm also appeared to be significantly higher than the KNN algorithm’s false-positive (4.04473%) and false-negative (6.24672%) values, while also appearing to be higher than the KNN algorithm’s false-positive (5.03173%) and false-negative (7.21573%) values. Hence, the results proved that the forward-propagation workflow of the CNN algorithm’s performance concerning accuracy, precision, false positive and false negative was better than that of the KNN and SVM algorithms as far as the detection of malicious information in the network packet datasets was concerned, which also helped establish the novelty of this research. It is also concluded that this malicious packet detection mechanism could be extended in real-time traffic data as an incident response system. There is a chance that if any performance comparison is carried out, this could be a solution using unsupervised learning.

In order to further improve the performance measures, this work can be implemented using variations of deep-learning algorithms like RNN or ANN or with modifications in the CNN algorithms to minimize the time complexity. Furthermore, a real-time dataset can be used by establishing and pumping relevant packets to be traced. In addition, automated false positives, false negatives and recall performance measures could be inserted as supplementary performance measure parameters alongside the accuracy, precision, and false-positive and false-negative parameters. It is also necessary to develop a model for network traffic classification with the combined effect of CNN and RNN in real-time traffic communications, as is discussed in work by Lopez-Martin et al. [36]. Furthermore, web attack identification methods might be implemented with the CNN algorithm, as discussed by Pang et al. using a deep network [30], Kamarudin et al. [29] using a logit boost-based algorithm, and Kim et al. [24]. This proposed work also could be extended for WSN-based surveillance communication [42,43,44] for better efficiency and throughput.

## Figures and Tables

**Table 1:** Method and applications of malicious information identification.

S. No	Identification Approach	Learning Algorithm	Application	Performance Parameters	Benefits
1	Users’ time-series data from network packets from cicids2017 dataset [3]	DL algorithm	Malicious behavior identification	Accuracy and recall, false positive	Time-series-based detection is applicable for real-time detection
2	Malicious packet identification techniques [6,7,8]	ML algorithm	Malicious traffic identification	Accuracy	ML enabled is essential for multimedia network traffic
3	Botnet detection procedure using flow-based detection [9,11]	Supervised ML algorithm	Malicious packet information identification	Precise time and accuracy	Applicable for chat bot in real time

**Table 2:** Work related to various methods for detecting malicious information.

S. No	Method	Application	Datasets Used	Benefits
1	Network-based anomaly detection [3]	IoT traffic	Commercial IoT dataset	It will be most suited for malicious user detection with IoT
2	CNN-based malware traffic detection [1]	Network traffic classification	Network traffic data images	Image-based malicious information can be detected with CNN
3	RNN model [7,8]	Packet feature extraction	Network traffic	Packet feature extraction is an easy way to detect malicious information with RNN
4	ML-based model [16]	Malicious packet identification	Network traffic	An emerging application for network traffic
5	CNN-based packet flow [17]	Malicious packet based on packet features	Flow packet dataset	Flow packet dataset is best suited to detect malicious information with CNN
6	Encrypted malicious packet [18]	Encrypted malicious packet identification	Real-time encrypted packet	Advanced technique without disclosing intended information

**Table 3:** Maliciousness confusion matrix.

Identification Approach	Has Malicious Information	Does Not Have Malicious Information
Identified as malicious	True positive	False positive
Not identified as malicious	False negative	True negative

**Table 4:** Iteration values of performance measuring parameters.

Algorithm	Accuracy	Precision	FPR	FNR
CNN	98.604	98.747	1.365	4.106
	97.754	99.497	1.615	2.966
	99.704	98.737	2.375	4.74
	97.794	99.512	1.6	2.888
	99.714	98.696	2.416	4.6
	99.874	99.609	1.503	3.872
	99.674	98.774	2.338	4.917
	98.894	99.531	1.581	2.639
	97.694	98.671	2.441	2.613
	99.984	99.666	1.446	1.648
	97.644	98.531	2.581	3.482
KNN	95.489	96.635	4.472	7.219
	96.641	96.385	3.732	5.079
	95.591	95.625	4.482	6.853
	94.68	96.4	3.712	6.001
	95.6	95.584	4.532	6.713
	94.762	96.497	3.612	5.985
	95.559	95.662	4.452	7.03
	94.776	96.419	3.692	5.752
	95.581	95.559	4.552	6.726
	96.866	95.554	3.562	5.761
	95.528	95.419	3.692	5.595

**Table 5:** Group statistics (mean, std. deviation, std. error mean) for performance measuring parameters.

Group Statistics					
	Algorithm	N	Mean	Std. Deviation	Std. Error Mean
Accuracy	CNN	11	98.848	0.982	0.296
	KNN	11	95.551	0.700	0.211
Precision	CNN	11	99.088	0.460	0.1389
	KNN	11	95.976	0.478	0.144
False positive	CNN	11	1.932	0.484	0.1461
	KNN	11	4.044	0.437	0.131
False negative	CNN	11	3.497	1.043	0.314
	KNN	11	6.24672	0.690	0.208

**Table 6:** Independent sample test for CNN and KNN for significance calculation statistics.

Independent Samples Test										
		Levene’s Test for Equality of Variances		*t*-test for Equality of Means						
		F	Sig.	t	df	Sig. (2-tailed)	Mean Difference	Std. Error Difference	95% Confidence Interval of the Difference	
									Lower	Upper
Accuracy	Equal Variances Assumed	4.472	0.047	9.059	20	000	3.296	0.363	2.537	4.055
	Equal Variances Not Assumed			9.059	18.077	000	3.296	0.363	2.532	4.060
Precision	Equal Variances Assumed	0.138	0.714	15.540	20	000	3.112	0.200	2.694	3.529
	Equal Variances Not Assumed			15.540	19.973	000	3.112	0.200	2.694	3.529
False positive	Equal Variances Assumed	1.201	0.286	−10.728	20	000	−2.111	0.196	−2.522	−1.701
	Equal Variances Not Assumed			−10.728	19.790	000	−2.111	0.196	−2.522	−1.700
False negative	Equal Variances Assumed	2.175	0.156	−7.289	20	000	−2.749	0.377	−3.536	−1.962
	Equal Variances Not Assumed			−7.289	17.355	000	−2.749	0.377	−3.543	−1.954

**Table 7:** 11-epoch performance values of accuracy, precision, false positive and false negative.

Algorithm	Accuracy	Precision	FPR	FNR
CNN	98.604	98.747	1.365	4.106
	97.754	99.497	1.615	2.966
	99.704	98.737	2.375	4.74
	97.794	99.512	1.6	2.888
	99.714	98.696	2.416	4.6
	99.874	99.609	1.503	3.872
	99.674	98.774	2.338	4.917
	98.894	99.531	1.581	2.639
	97.694	98.671	2.441	2.613
	99.984	99.666	1.446	1.648
	97.644	98.531	2.581	3.482
SVM	94.37	95.246	5.459	8.188
	95.522	94.996	4.719	6.048
	94.472	94.236	5.469	7.822
	93.561	95.011	4.699	6.97
	94.481	94.195	5.519	7.682
	93.643	95.108	4.599	6.954
	94.44	94.273	5.439	7.999
	93.657	95.03	4.679	6.721
	94.462	94.17	5.539	7.695
	95.747	94.165	4.549	6.73
	94.409	94.03	4.679	6.564

**Table 8:** Group statistics comparison of the CNN and SVM algorithms for performance measures.

Group Statistics					
	Algorithm	N	Mean	Std. Deviation	Std. Error Mean
Accuracy	CNN	11	98.84855	0.982796	0.296324
	SVM	11	94.43319	0.700602	0.211240
Precision	CNN	11	99.08827	0.460946	0.138980
	SVM	11	94.58727	0.478196	0.144181
False positive	CNN	11	1.93287	0.484868	0.146193
	SVM	11	5.03173	0.437266	0.131841
False negative	CNN	11	3.49736	1.043027	0.314484
	SVM	11	7.21573	0.690754	0.208270

**Table 9:** CNN and SVM algorithm-based malicious information identification independent samples test.

Independent Samples Test										
		Levene’s Test for Equality of Variances		*t*-Test for Equality of Means						
		F	Sig.	t	df	Sig. (2-Tailed)	Mean Difference	Std. Error Difference	95% Confidence Interval of the Difference	
									Lower	Upper
Accuracy	Equal variances assumed	4.472	0.047	9.059	20	000	3.296563	0.363903	2.537476	4.055650
	Equal variances not assumed			9.059	18.077	000	3.296563	0.36390	2.532266	4.060860
Precision	Equal variances assumed	0.138	0.714	15.540	20	000	3.112078	0.20026	2.694333	3.529823
	Equal variances not assumed			15.540	19.973	000	3.112078	0.20026	2.694297	3.529859
False positive	Equal variances assumed	1.201	0.286	−10.728	20	000	−2.111853	0.19686	−2.522500	−1.701207
	Equal variances not assumed			−10.728	19.790	000	−2.111853	0.19686	−2.522779	−1.700928
False negative	Equal variances assumed	2.175	0.156	−7.289	20	000	−2.749358	0.37717	−3.536135	−1.962582
	Equal variances not assumed			−7.289	17.355	000	−2.749358	0.37717	−3.543894	−1.954823

## Data Availability

Not applicable.

## References

[1] Cheng R. (2017). D 2 PI: Identifying Malware through Deep Packet Inspection with Deep Learning. Corpus ID: 53062187. https://www.semanticscholar.org/paper/D-2-PI-%3A-Identifying-Malware-through-Deep-Packet-Cheng/96011b826e2eba80c5e676de687114e9f88dcebe#citing-papers.

[2] Karasek D.Y., Kim J., Kemmoe V.Y., Bhuiyan M.Z.A., Cho S., Son J. SuperB: Superior Behavior-based Anomaly Detection Defining Authorized Users’ Traffic Patterns. Proceedings of the International Conference on Computer Communications and Networks (ICCCN).

[3] Thamilarasu G., Chawla S. (2019). Towards Deep-Learning-Driven Intrusion Detection for the Internet of Things. Sensors.

[4] Riyaz B., Ganapathy S. (2020). A deep learning approach for effective intrusion detection in wireless networks using CNN. Soft. Comput..

[5] Lopez-Martin M., Carro B., Sanchez-Esguevillas A., Lloret J. (2017). Network Traffic Classifier With Convolutional and Recurrent Neural Networks for Internet of Things. IEEE Access.

[6] Meidan Y., Bohadana M., Mathov Y., Mirsky Y., Shabtai A., Breitenbacher D. (2018). N-BaIoT—Network-Based Detection of IoT Botnet Attacks Using Deep Autoencoders. IEEE Pervasive Comput..

[7] Yin C., Zhu Y., Fei J., He X. (2017). A deep learning approach for intrusion detection using recurrent neural networks. IEEE Access.

[8] Li C., Wang J., Ye X. (2018). Using a Recurrent Neural Network and Restricted Boltzmann Machines for Malicious Traffic Detection. NeuroQuantology.

[9] Shinan K., Alsubhi K., Alzahrani A., Ashraf M.U. (2021). Machine Learning-Based Botnet Detection in Software-Defined Network: A Systematic Review. Symmetry.

[10] Gilmore C., Haydaman J. Anomaly detection and machine learning methods for network intrusion detection: An industrially focused literature review. Proceedings of the International Conference on Security and Management (SAM).

[11] Hwang R.-H., Peng M.-C., Nguyen V.-L., Chang Y.-L. (2019). An LSTM-Based Deep Learning Approach for Classifying Malicious Traffic at the Packet Level. J. Appl. Sci..

[12] Kang M.-J., Kang J.-W. (2016). Intrusion Detection System Using Deep Neural Network for In-Vehicle Network Security. PLoS ONE.

[13] Ahmed I., Lhee K.S. (2011). Classification of packet contents for malware detection. J. Comput. Virol..

[14] Wang W., Zhu M., Zeng X., Ye X., Sheng Y. Malware Traffic Classification Using Convolutional Neural Networks for Representation Learning. Proceedings of the International Conference on Information Networking.

[15] Publicly Available Library of Malwares (VX Heavens). http://vx.netlux.org/.

[16] Gibert D., Mateu C., Planes J. (2020). The rise of machine learning for detection and classification of malware: Research developments, trends and challenges. J. Netw. Comput. Appl..

[17] Yeo M., Koo Y., Yoon Y., Hwang T., Ryu J., Song J., Park C. Flow-based malware detection using convolutional neural network. Proceedings of the 2018 International Conference on Information Networking (ICOIN).

[18] de Lucia M.J., Cotton C. Detection of Encrypted Malicious Network Traffic using Machine Learning. Proceedings of the IEEE Military Communications Conference (MILCOM).

[19] Marín G., Caasas P., Capdehourat G. (2021). Deepmal-deep learning models for malware traffic detection and classification. Data Science–Analytics and Applications.

[20] Soe Y.N., Feng Y., Santosa P.I., Hartanto R., Sakurai K. (2020). Machine Learning-Based IoT-Botnet Attack Detection with Sequential Architecture. Sensors.

[21] Ronen R., Radu M., Feuerstein C., Yom-Tov E., Ahmadi M. (2018). Microsoft Malware Classification Challenge. arXiv.

[22] Shone N., Ngoc T.N., Phai V.D., Shi Q. (2018). A Deep Learning Approach to Network Intrusion Detection. IEEE Trans. Emerg. Top. Comput. Intell..

[23] Sharafaldin I., Gharib A., Lashkari A.H., Ghorbani A. (2017). Towards a reliable intrusion detection benchmark dataset. Softw. Netw..

[24] Kim T.-Y., Cho S.-B. (2018). Web traffic anomaly detection using c-lstm neural networks. Expert Syst. Appl..

[25] Erfani S.M., Rajasegarar S., Karunasekera S., Leckie C. (2016). High-dimensional and large-scale anomaly detection using a linear one-class SVM with deep learning. Pattern Recognit..

[26] Herrmann D., Wendolsky R., Federrath H. Website Fingerprinting: Attacking Popular Privacy Enhancing Technologies with the Multinomial NaïVe-bayes Classifier. Proceedings of the 2009 ACM Workshop on Cloud Computing Security.

[27] Reddy S., Shyam G.K. (2020). A machine learning based attack detection and mitigation using a secure SaaS framework. J. King Saud Univ.-Comput. Inf. Sci..

[28] Lotfollahi M., Siavoshani M.J., Zade R.S.H., Saberian M. (2019). Deep packet: A novel approach for encrypted traffic classification using deep learning. Soft Comput..

[29] Kamarudin M.H., Maple C., Watson T., Safa N.S. (2017). A logitboostbased algorithm for detecting known and unknown web attacks. IEEE Access.

[30] Pang G., Shen C., Jin H., van den Hengel A. (2020). Deep weaklysupervised anomaly detection. arXiv.

[31] Aamir M., Ali T., Shaf A., Irfan M., Saleem M.Q. (2020). ML-DCNNet: Multi-level Deep Convolutional Neural Network for Facial Expression Recognition and Intensity Estimation. Arab. J. Sci. Eng..

[32] Aamir M., Irfan M., Ali T., Ali G., Shaf A., S A.S., Al-Beshri A., Alasbali T., Mahnashi M.H. (2020). An Adoptive Threshold-Based Multi-Level Deep Convolutional Neural Network for Glaucoma Eye Disease Detection and Classification. Diagnostics.

[33] Aamir M., Ali T., Irfan M., Shaf A., Azam M., Glowacz A., Brumercik F., Glowacz W., Alqhtani S., Rahman S. (2021). Natural Disasters Intensity Analysis and Classification Based on Multispectral Images Using Multi-Layered Deep Convolutional Neural Network. Sensors.

[34] Fan G.-F., Zhang L.-Z., Yu M., Hong W.-C., Dong S.-Q. (2022). Applications of Random forest in multivariable response surface for short-term load forecasting. Int. J. Electr. Power Energy Syst..

[35] Ding M., Tian H. (2016). Pca-based network traffic anomaly detection. Tsinghua Sci. Technol..

[36] Wang W., Huang H., Zhang L., Su C. (2021). Secure and efficient mutual authentication protocol for smart grid under blockchain. Peer--Peer Netw. Appl..

[37] Zhang L., Peng M., Wang W., Jin Z., Su Y., Chen H. (2021). Secure and efficient data storage and sharing scheme for blockchain-based mobile-edge computing. Trans. Emerg. Telecommun. Technol..

[38] Zhang L., Zou Y., Wang W., Jin Z., Su Y., Chen H. (2021). Resource allocation and trust computing for blockchain-enabled edge computing system. Comput. Secur..

[39] Wang W., Xu H., Alazab M., Gadekallu T.R., Han Z., Su C. (2021). Blockchain-Based Reliable and Efficient Certificateless Signature for IIoT Devices. IEEE Trans. Ind. Inform..

[40] Lian Z., Wang W., Su C. COFEL: Communication-Efficient and Optimized Federated Learning with Local Differential Privacy. Proceedings of the ICC 2021-IEEE International Conference on Communications.

[41] Wang W., Qiu C., Yin Z., Srivastava G., Gadekallu T.R., Alsolami F., Su C. (2021). Blockchain and PUF-based Lightweight Authentication Protocol for Wireless Medical Sensor Networks. IEEE Internet Things J..

[42] Adimoolam M., Sugumaran M., Rajesh R.S. (2018). Efficient encryption algorithm for video data storage. Int. J. Inf. Comput. Sci..

[43] Adimoolam M., Sugumaran M., Rajesh R.S. (2018). A Novel Efficient Redundancy free Data Communication Model for Intelligent Surveillance System in WSN. J. Adv. Res. Dyn. Control Syst..

[44] Adimoolam M., Sugumaran M., Rajesh R.S. (2018). A Novel Efficient Clustering and Secure Data Transmission Model for Spatiotemporal Data in WSN. Int. J. Pure Appl. Math..

